# Comparison of the sugar and organic acid components of seventeen table grape varieties produced in Ankara (Türkiye): a study over two consecutive seasons

**DOI:** 10.3389/fpls.2024.1321210

**Published:** 2024-03-08

**Authors:** Birhan Kunter, Osman Batur Unal, Sıddık Keskin, Harlene Hatterman-Valenti, Ozkan Kaya

**Affiliations:** ^1^ Department of Horticulture, Faculty of Agriculture, Ankara University, Ankara, Türkiye; ^2^ Graduate School of Natural and Applied Sciences, Ankara University, Ankara, Türkiye; ^3^ Department of Biostatistics, Faculty of Medicine, Van Yuzuncu Yıl University, Van, Türkiye; ^4^ Department of Plant Sciences, North Dakota State University, Fargo, ND, United States; ^5^ Erzincan Horticultural Research Institute, Republic of Turkey Ministry of Agriculture and Forestry, Erzincan, Türkiye

**Keywords:** grapevine, table grape variety, sugar, organic acid, HPLC

## Abstract

Sugars and organic acids not only have a significant impact on taste balance and sensory acceptance by consumers but also play a crucial role in the chemical equilibrium of grape juices and wines. Therefore, this study aimed to quantify the content and composition of sugars and organic acids in 17 grape varieties over two consecutive years using high-performance liquid chromatography. The variability in all the parameters studied was strongly influenced by both the grape cultivars and specific years (*p* ≤ 0.05). In grape berries, the primary sugars identified were fructose and glucose, which ranged from 6.50 to 11.10 g/L and from 5.83 to 12.12 g/L, respectively, over the two years. However, sucrose was not detected in any of the grape varieties examined. For the two respective years, the highest titratable acidity (TA) was found in Tekirdağ Çekirdeksizi (TeCe) (0.89 and 0.90 g/L), while the lowest was detected in Victoria (Vi) (0.48 and 0.51 g/L). Total soluble solids (TSS) peaked in Horoz Karası (HoKA) (21.90 °Brix), whereas it reached its lowest point in Big Perlon (BiPe) (14.1 °Brix). The tartaric acid content in the grape berries, ranging from 1.48 to 10.33 g/L for the two years, exhibited similar characteristics to malic acid, which ranged from 1.09 to 9.62 g/L and from 1.03 to 9.68 g/L for the two respective years. The succinic, malic, tartaric, citric, and oxalic acid contents were notably higher in the Kyoho (Ky) variety than in the other varieties. When examining the dendrogram of the contents of organic acid and sugar for similarities, it was evident that 16 out of the 17 grape varieties had a high degree of similarity, except for Alphonse Lavallée (AlLa) and HoKa. The similarity levels among the varieties ranged from 99.49% to 72.36%. The highest similarity (99.49%) was observed between the AlLa and Barış (Ba) varieties. The lowest similarity was observed among the AlLa, HoKa, and Ky varieties. In summary, this study underscores that certain table grape varieties grown in Ankara exhibit significant variations in valuable organic acids and sugars, which are associated with potential health benefits when considering human consumption.

## 1 Introduction

Grapes (*Vitis vinifera* L.), which have been cultivated since ancient times, play a pivotal role in consumable and processable agricultural products (Patil et al., 1995; Keller, 2020). They are cherished for their positive impact on human health and substantial economic significance (Ivanova-Petropulos et al., 2015). Turkey plays a significant role in this industry, ranking sixth in the world in terms of grape production in 2020, as reported by the FAO, 2021. In Turkey, the utilization of grapes is diverse: approximately 3% is used for wine production, 33% is dried to produce raisins, 27% is sold as table grapes, and 37% is processed into traditional products such as pekmez (boiled concentrate juice), kofter, vinegar, sausage, and grape juice (Keskin et al., 2019; Keskin et al., 2021). A comprehensive understanding of the biochemical composition of this valuable product, along with its diverse consumption patterns, is of interest to both researchers and consumers. Organic acids are widely recognized as primary metabolites in grape berries, and the profile and concentration of these compounds have been underscored as pivotal factors influencing the processing and chemical makeup of wine and grape juices (Coelho et al., 2018; Mendes Ferreira and Mendes-Faia, 2020; Dutra et al., 2021). The presence of acids, such as lactic, malic, and tartaric acid, significantly affects the chemical stability, pH, taste equilibrium, and aroma, which, in turn, contribute to the overall flavor of grape-based products (Ricardo-da-Silva et al., 2015). Sugars and organic acids, quantified through total soluble solids (TSS) and titratable acidity (TA), are closely associated with the flavor of grape berries and play a crucial role in consumer preferences (Shiraishi et al., 2010).

Furthermore, the content and composition of organic acids and sugars in grapes are vital for the breeding and selection of new grape varieties (Liu et al., 2006). Consequently, the nature and quantity of the sugars and acids in grapes have been extensively studied in recent decades (Liu et al., 2006; Shiraishi et al., 2010; Dutra et al., 2021; Keskin et al., 2021). Glucose and fructose are the dominant sugars in the majority of grape varieties, whereas sucrose is present only in trace amounts. Nevertheless, certain hybrids exhibit higher sucrose levels because of their genetic background (Lott and Barrett, 1967; Carroll et al., 1971). The starch concentration in grape berries is almost negligible, with fructose and glucose present in roughly equal quantities, whereas sucrose contributes less than 1% (Conde et al., 2015). Organic acids are found in smaller quantities than sugars in grape berries, with malic and tartaric acids being the primary ones, usually accounting for over 90% of the total acids (Muñoz-Robredo et al., 2011). Succinic and citric acids are also present in lower proportions (Soyer et al., 2003; Liu et al., 2006). The ratio of malic acid to tartaric acid varies among grape varieties and is influenced by their genetic background (Kanellis and Roubelakis-Angelakis, 1993). Moreover, factors such as geographical location, climate, viticultural practices, ripening stage, and grape variety influence the organoleptic quality of grapes (Yamamoto et al., 2015; Granato et al., 2016). The most commercially significant grape varieties are derived from *V. vinifera*, and hybrids formed between *V. vinifera* and *Vitis labrusca* grapes are used in winemaking and are consumed as raisins and table grapes. In contrast, varieties resulting from hybrids of *V. vinifera* and *V. labrusca* are primarily cultivated for table grapes because of their enhanced resistance to diseases in regions with high humidity and substantial precipitation during the growing season (Tonietto and Carbonneau, 2004). The adaptation of grape varieties to local environmental conditions is influenced by the specific climate and soil characteristics of vineyard regions (Fraga et al., 2012). The Ankara region, characterized by a continental climate, poses challenges for the production of some table grape varieties in terms of soil and climatic conditions. Nevertheless, there has been an increasing trend in table grape production in this region in recent years, making the assessment of variety suitability a matter of increasing importance.

Although the composition of organic acids and sugars in table grape varieties is influenced by their genetic makeup, variations may arise due to growing conditions. To contribute to the existing body of knowledge, it is essential to investigate the content and the composition of organic acids and sugars in grape varieties cultivated under the unique conditions in Ankara. To date, no information regarding the sugar and organic acid contents and the composition of grapes grown in Ankara is available, except for a limited number of grape varieties. Therefore, this study aimed to examine the organic acid and sugar composition of 17 grape varieties cultivated in the Ankara region and to provide valuable insights for future breeding programs targeting the enhancement of berry quality in grapes.

## 2 Materials and methods

### 2.1 Plant material

In this study, we examined 17 grape cultivars consisting of 15 vinifera varieties—Barış (Ba), Horoz Karası (HoKa), Köhnü (Ko), Tekirdağ Çekirdeksizi (TeCe), Trakya İlkeren (TrIk), Uslu (Us), Yalova İncisi (YaIn), Yalova Misketi (YaMi), Alphonse Lavallée (AlLa), Big Perlon (BiPe), Lival (Li), Prima (Pr), Ribol (Ri), Royal (Ro), and Victoria (Vi); one labrusca grape variety, Isabella (Is); and one *V. labrusca × V. vinifera* hybrid grape variety, Kyoho (Ky). These varieties were studied over two consecutive years (2018–2019). All samples were collected from an experimental vineyard located in Kalecik, Ankara (40° 06′ N, 33° 25′ E, 670 m above sea level). These vines were initially planted in the spring of 2005 and trained in cordon. They were spaced 1.5 m apart within the row and 3.0 m apart between rows, with an east–west row orientation. The same management practices, including pruning, fertilization, soil management, irrigation, and disease control, were consistently applied in the vineyard. Berries from different positions within each cluster were mixed and analyzed. Seeds from berries of the seeded varieties were removed immediately. A random set of berries per replicate was collected and frozen at −20°C for subsequent analysis. All clusters from the six replicates were manually destemmed and crushed in the laboratory. Ripening parameters such as the TA, TSS (in degrees Brix), and pH were determined.

### 2.2 Organic acid and sugar analysis

The composition of organic acids and sugars was analyzed using an Agilent 1100 Series G1362A RID-HPLC system. To determine the acids, we modified the method described by Bevilacqua and Califano (1989). For this analysis, 1 g of each sample was placed in centrifuge tubes and 20 mL of 0.009 N H_2_SO_4_ was added. After homogenization with a homogenizer (Heidolph, Schwabach, Germany), the samples were mixed for 1 h on a shaker (Heidolph unimax 1010, Germany) and then centrifuged at 15,000 rpm for 15 min. The aqueous fraction separated in the centrifuge was first filtered using a coarse filter paper and then twice through a 0.45-µm membrane filter (Millipore Millex-HV Hydrophilic PVDF; Millipore, St. Louis, MO, USA). Finally, the samples were passed through a C18 Sep-Pak cartridge. The organic acid composition was analyzed using an Aminex HPX-87 H 300 mm × 7.8 mm column (BioRad, Hercules, CA, USA) and an Agilent 1100 model G1315B DAD detector HPLC system. Readings were taken at a wavelength of 214 nm, with the column furnace temperature set at 30°C during the analysis. The mobile phase in the system was 0.009 N H_2_SO_4_, filtered through a 0.45-µm membrane filter with a flow rate of 0.4 mL/min.

To determine the sugars, we adapted the method described by Liu et al. (2006). Approximately 5 g of each sample was combined with 40 mL of ultrapure water. This solution was transferred into a 100-mL measuring flask containing 25 mL of methanol and the volume adjusted to 100 mL with ultrapure water. After centrifugation at 10,000 rpm for 10 min, the filtrates were passed through a 0.45-µm diameter membrane filter and collected in vials. The obtained extracts were directly injected into an Agilent 1100 Series model G1362A HPLC (Agilent Technologies) device with a refractive index detector (RID) and then transferred into a Hypersil GOLD Amino 5 µm, 250 mm × 4.6 mm column (Thermo Fisher Scientific, Waltham, MA, USA). The column furnace temperature was set to 30°C during the analysis, with the mobile phase being acetonitrile/water (80:20) flowing at a rate of 1.3 mL/min.

### 2.3 Statistical analysis

The data for each variety in each year were calculated as the average of three replications. Variance analysis, specifically the one-way analysis of variance (ANOVA), was carried out using SPSS (ver. 21) and MINITAB (ver. 19) statistical software. Differences were compared using Duncan’s test (*p* ≤ 0.05).

## 3 Results

For grape cultivation in Kalecik, the harvesting process was divided into three distinct periods based on the maturation stages of the grape varieties. The first category encompassed early-season varieties, including TeCe, TrIk, Us, YaIn, YaMi, Li, Pr, and Ky. The mid-season group consisted of two varieties, HoKa and Vi. Lastly, the late-season group comprised seven varieties: Ba, Ko, AlLa, BiPe, Ri, Ro, and Is. During the harvest period, the TSS content ranged from 14.1°Brix (BiPe) to 21.0°Brix (YaMi). Among the early ripening varieties, YaMi exhibited the highest TSS content at 21.0°Brix, whereas TeCe exhibited the lowest content at 16.2°Brix. In the mid-season varieties, HoKa and Vi displayed TSS values of 19.4 and 19.6°Brix, respectively. Among the late-season ripening varieties, Is had the highest TSS at 20.1°Brix, whereas BiPe had the lowest at 14.1°Brix (**Table 1**). It is worth noting that, for both years, significant differences (*p* ≤ 0.05) were detected in the TSS values among the grape varieties. In this study, focusing on grape cultivars under Kalecik conditions, we observed notable differences in key viticultural metrics across two consecutive years. First, a significant variation in the °Brix values was recorded between years. Remarkably, all grape varieties consistently achieved a °Brix content exceeding 14 °Brix. Furthermore, the analysis revealed substantial differences in the TA content among the grape varieties and between the two years. Specifically, the TeCe variety exhibited the highest TA content for both years, recorded at 0.89 and 0.90 g/L, respectively. In contrast, the Vi variety showed the lowest TA values, marked at 0.48 and 0.51 g/L in the respective years. Regarding the total soluble solids-to–total acidity (TSS/TA) ratio, a critical indicator of grape quality, significant variations were found within our study period. In 2018, the TSS/TA ratio ranged from 18.45 (in TeCe) to 38.65 (in Vi). The following year (2019) recorded a range of 17.36 (in TrIk) to 45.26 (in Is). In addition, the pH values of the grapes varied. In 2018, the highest pH recorded was 3.6 (Us and YaIn), while the lowest was 3.0 (TeCe). In 2019, the highest pH values were observed in Us, YaIn, and Ri (pH 3.7) and the lowest observed in TeCe and TrIk (pH 3.4) (**Table 1**).

**Table 1:** Classic analysis and sugar contents of different grape varieties (mean).

Variety	TSS (^o^Brix)		TA (g/L)			^o^Brix/TA	pH	Glucose (g/100 g)			Fructose (g/100g)			Glucose/Fructose (g/100g)			∑ Sugars (g/100 g)	
	2018	2019	2018		2019	2018	2019	2018	2019	2018	2019	2018		2019	2018	2019	2018	2019
**Ba**	18.30^bA^	15.20^deB^	0.68^cA^		0.56^eB^	26.75^bc^	26.90^c^	3.4^b^	3.6^a^	9.30^a-d^	9.17^b-d^	9.29^cd^		9.69^c^	1.00^c-e^	0.94^ef^	18.59^bc^	18.87^b-d^
**HoKa**	19.40^abB^	21.90^aA^	0.59^d^		0.65^c^	32.49^b^	33.48^b^	3.4^b^	3.6^a^	9.40^a-d^	8.89^c-e^	10.31^bA^		8.51^d-fB^	0.91^f B^	1.04^bcA^	19.71^bc^	17.40^d-g^
**Ko**	17.20^cB^	19.10^bA^	0.51^eB^		0.64^cA^	33.85^b^	29.65^c^	3.2^cB^	3.6^aA^	9.45^a-d^	11.10^a^	10.25^bB^		12.12^aA^	0.92^ef^	0.91^f^	19.71^bc^	23.22^a^
**TeCe**	16.20^dB^	18.10^bcA^	0.89^a^		0.90^a^	18.45^d^	20.11^d^	3.0^dB^	3.4^bA^	7.06^ıB^	11.08^aA^	7.86^eB^		11.08^bA^	0.89^f^	1.00^b-e^	14.92^e^	22.17^a^
**TrIk**	16.30^dA^	14.00^eB^	0.70^bcB^		0.81^bA^	23.15^cA^	17.36^eB^	3.3^bc^	3.4^b^	9.57^a-dA^	6.20^hB^	9.25^cdA^		5.83^kB^	1.03^b-d^	1.06^ab^	18.82^bc^	12.03^j^
**Us**	19.00^abA^	15.60^dB^	0.61^dB^		0.74^bA^	31.19^bA^	21.25^dB^	3.6^a^	3.7^a^	9.75^a-c^	10.00^b^	10.06^bc^		9.73^c^	0.96^d-fB^	1.02^bdA^	19.82^b^	19.73^b^
**YaIn**	17.20^cA^	13.90^eB^	0.54^eB^		0.64^cA^	31.97^bA^	21.85^dB^	3.6^a^	3.7^a^	8.38^e-g^	9.30^bc^	8.22^eB^		9.82^cA^	1.01^b-d^	0.94^ef^	16.61^d^	19.12^bc^
**YaMi**	21.00^a^	20.50^ab^	0.67^cB^		0.75^bA^	31.48^b^	27.47^c^	3.4^b^	3.6^a^	6.94^ıB^	8.79^c-eA^	6.35^f B^		9.03^cdA^	1.09^abA^	0.97^c-fB^	13.29^f^	17.82^c-f^
**AlLa**	14.60^eB^	17.70^cA^	0.66^cA^		0.62^dB^	22.15^cB^	28.68^cA^	3.4^b^	3.6^a^	8.69^c-e^	8.24^d-f^	9.43^cd^		8.56^de^	0.92^ef^	0.96^d-f^	18.12^c^	16.81^e-h^
**BiPe**	14.10^e^	14.50^e^	0.60^dB^		0.67^cA^	23.46^c^	21.54^d^	3.2^cB^	3.5^abA^	7.58^g-ı^	6.50^h^	6.63^f^		6.53^ı-k^	1.14^a^	0.99^b-e^	14.21^ef^	13.03^ıj^
**Li**	14.70^eB^	16.30^dA^	0.73^bA^		0.60^eB^	20.27^cB^	26.89^cA^	3.2^c^	3.5^ab^	8.90^b-e^	9.18^b-d^	9.69^b-d^		9.26^cd^	0.91^fB^	0.99^beA^	18.59^bc^	18.44^b-e^
**Pr**	17.30^c^	18.80^bc^	0.69^cB^		0.79^bA^	25.14^bc^	23.55^d^	3.3^bcB^	3.6^aA^	8.99^b-e^	8.14^ef^	9.27^cdA^		7.81^e-gB^	0.97^d-f^	1.04^bc^	18.26^bc^	15.95^gh^
**Ri**	18.60^b^	19.80^ab^	0.63^d^		0.67^c^	29.52^b^	29.50^c^	3.3^bcB^	3.7^aA^	7.12^hı^	6.81^gh^	7.45^e^		6.90^h-j^	0.95^d-f^	0.98^c-f^	14.57^ef^	13.71^ı^
**Ro**	18.10^bA^	15.20^deB^	0.63^dB^		0.68^cA^	28.73^bA^	22.15^dB^	3.5^ab^	3.6^a^	10.33^aA^	8.09^efB^	11.08^aA^		7.25^f-hB^	0.93^efB^	1.11^aA^	21.42^a^	15.35^h^
**Vi**	19.60^abA^	17.20^cB^	0.51^e^		0.48^f^	38.65^a^	35.75^b^	3.5^ab^	3.6^a^	7.99^f-h^	8.37^c-f^	7.54^e^		8.05^d-f^	1.05^bc^	1.03^b-d^	15.54^de^	16.42^f-h^
**Is**	20.10^a^	21.50^a^	0.72^bA^		0.48^fB^	27.99^bc^	45.26^a^	3.2^c^	3.6^a^	9.57^a-cA^	7.63f^gB^	9.01^dA^		7.68^f-hB^	1.06^bc^	0.99^b-e^	18.59^bc^	15.32^h^
**Ky**	17.40^cB^	20.90^aA^	0.78^a^		0.79^b^	22.30^c^	26.25^c^	3.3^bc^	3.5^ab^	9.93^abA^	6.67^hB^	9.65^b-dA^		6.39^jkB^	1.02^b-d^	1.04^bc^	19.58^bc^	13.06^ıj^

Different lowercase letters within the columns indicate significant differences in the sugar contents of the grape varieties at the p ≤ 0.05 level by Duncan’s LSD multiple comparison test. Different capital letters within the lines indicate significant differences between years in the sugar contents of the grape varieties at the p ≤ 0.05 level by Duncan’s LSD multiple comparison test.

TSS, total soluble solids; TA, titratable acidity.

Our analysis of the fructose, glucose, fructose/glucose ratio, and total sugar content in the various grape varieties revealed significant differences (*p* ≤ 0.05). In terms of sugar composition, grapes at the end of ripening predominantly contained glucose and fructose, making up approximately 99% of the total sugar content. For the year 2018, the glucose levels ranged from 6.94 to 10.33 g/100 g, while those in 2019 varied from 6.20 to 11.10 g/100 g across different varieties. Ro exhibited the highest average fructose content in 2018, followed by HoKa, Ko, Us, Li, and Ky. In contrast, Ko exhibited the highest fructose accumulation in 2019, reaching 12.12 g/100 g. The glucose and fructose contents of all the grape varieties were similar. Among the varieties studied, Ba, Ko, Us, and Li consistently showed the highest total sugar content for both years (**Table 1**). In this study, we meticulously analyzed the concentrations of organic acids in the different grape varieties, which uncovered a remarkable range in their composition. The total organic acid content showed considerable variation across varieties, with the Ro variety exhibiting the lowest concentration at 3.95 g/L and the Ky variety the highest at 24.94 g/L. This variation was further underscored by the significant differences in the levels of tartaric acid among varieties. Furthermore, our study revealed a notable diversity in malic acid content, with 12 varieties falling within a consistent range. Four cultivars, including Ky, had notably higher levels, surpassing 3.40 g/L. The levels of citric acid also varied greatly, as did the concentrations of succinic acid, both displaying a wide range across the grape varieties studied in 2018 and 2019. An interesting outlier was the levels of oxalic acid, which were low across all varieties, except for Ky in which the levels were significantly higher, exceeding 0.5 g/L. In addition, the tartaric acid/malic acid ratio showed a broad spectrum of variation, indicating a diverse range of acid profiles in these grape varieties. The findings also pointed to substantial differences in the total organic acid concentrations between 2018 and 2019, ranging from 4.74 to 24.94 g/L and from 3.95 to 24.21 g/L, respectively (**Table 2**).

**Table 2:** Organic acid contents of the different grape varieties (mean).

Variety	Tartaric (g/L)		Malic (g/L)		Citric (g/L)		Succinic (g/L)		Oxalic (g/L)		Total (g/L)		Tar/Mal (g/L)	
	2018	2019	2018	2019	2018	2019	2018	2019	2018	2019	2018	2019	2018	2019
**Ba**	4.31^dc^	3.98^cd^	4.40^b^	4.09^bc^	0.32^fgB^	0.67^cA^	0.11^ı^	0.14^g^	0.01^e^	0.01^g^	9.18^c^	8.90^c^	1.03^de^	1.08^de^
**HoKa**	1.89^fg^	2.01^fg^	2.06^h-j^	2.13^fg^	0.31^g^	0.34^fg^	0.25^c^	0.25^c^	0.02^de^	0.01^g^	4.54^ı^	4.74^h^	0.91^de^	0.94^d-f^
**Ko**	3.46^e^	3.64^cd^	2.30^g-ı^	2.19^fg^	0.39^ef^	0.40^f^	0.11^ı^	0.11^g^	0.05^c^	0.05^c^	6.32^g^	6.39^f^	1.50^c^	1.66^b^
**TeCe**	1.98^fg^	1.94^fg^	3.03^ef^	3.12^de^	0.88^bB^	1.03^bA^	0.21^c-f^	0.24^cd^	0.01^e^	0.02^fg^	6.12^g^	6.35^f^	0.65^ef^	0.62^ef^
**TrIk**	3.90^c-e^	3.81^cd^	2.62^f-h^	2.49^ef^	0.61^c^	0.60^d^	0.17^e-h^	0.19^ef^	0.01^e^	0.01^g^	7.32^f^	7.10^e^	1.48^c^	1.53^bc^
**Us**	4.13^c-e^	4.23^c^	3.79^b-d^	3.75^b-d^	0.64^c^	0.68^c^	0.16^gh^	0.17^f^	0.02^de^	0.02^ef^	8.75^cd^	8.87^c^	1.09^d^	1.13^ef^
**YaIn**	3.78^deA^	3.14d^eB^	1.09^j^	1.03^h^	0.32^fg^	0.31^g^	0.24^cd^	0.24^c^	0.03^c-e^	0.02^fg^	5.46^hA^	4.75^hB^	3.47^a^	3.04^a^
**YaMi**	2.53^f^	2.61^ef^	3.19^d-f^	3.12^de^	0.29^g^	0.30^g^	0.17^efg^	0.19^ef^	0.04^cd^	0.05^c^	6.22^g^	6.27^fg^	0.79^d-f^	0.83^d-f^
**AlLa**	4.07^c-e^	4.16^c^	4.31^b^	4.23^b^	0.46^de^	0.47^e^	0.12^hı^	0.11^g^	0.03^c-e^	0.04^cd^	9.00^c^	9.02^bc^	0.94^de^	0.98^d-f^
**BiPe**	3.62^de^	4.18^c^	3.61^c-eB^	4.39^bA^	0.47^d^	0.49^e^	0.21^c-e^	0.20^d-f^	0.04^cd^	0.05^c^	7.96^eB^	9.31^bcA^	1.00^de^	0.95^d-f^
**Li**	3.48^eB^	3.93^cdA^	4.03^bc^	4.52^b^	0.52^d^	0.53^e^	0.19^e-g^	0.20^d-f^	0.02^de^	0.02^fg^	8.25^de^	9.21^bc^	0.86^de^	0.87^d-f^
**Pr**	4.53^c^	4.55^bc^	2.80^ef^	2.55^ef^	0.49^d^	0.49^e^	0.20^d-g^	0.21^c-e^	0.01^e^	0.02^ef^	8.05^e^	7.84^d^	1.62^c^	1.78^b^
**Ri**	1.48^g^	1.71^fg^	3.25^d-f^	3.32^c-e^	0.51^d^	0.50^e^	0.16^gh^	0.18^ef^	0.02^de^	0.02^ef^	5.44^h^	5.75^g^	0.45^f^	0.51^f^
**Ro**	5.58^bA^	1.41^gB^	1.83^ıj^	1.90^fg^	0.48^d^	0.47^e^	0.13^hı^	0.12^g^	0.04^cd^	0.03^de^	8.08^eA^	3.95^ıB^	3.04^bA^	0.74^d-fB^
**Vi**	2.51^f^	2.50^ef^	1.51^jk^	1.48^gh^	0.50^d^	0.52^e^	0.21^c-f^	0.23^cd^	0.01^e^	0.02^fg^	4.75^ı^	4.75^h^	1.66^c^	1.69^b^
**Is**	5.40^b^	5.28^b^	3.75^b-d^	3.11^de^	0.37^fg^	0.39^f^	0.56^bB^	0.65^bA^	0.09^b^	0.08^b^	10.18^b^	9.53^b^	1.44^c^	1.70^b^
**Ky**	10.33^aB^	10.99^aA^	9.62^a^	9.68^a^	2.60^a^	2.63^a^	1.12^a^	1.13^a^	0.52^a^	0.49^a^	24.21^a^	24.94^a^	1.07^d^	1.13^ef^

Different lowercase letters within the columns indicate significant differences in the organic acid contents of the grape varieties at the p ≤ 0.05 level by Duncan’s LSD multiple comparison test. Different capital letters within the lines indicate significant differences between years in the organic acid contents of the grape varieties at the p ≤ 0.05 level by Duncan’s LSD multiple comparison test.

Tar/Mal, tartaric-to-malic acid ratio.

**Figure 1:**
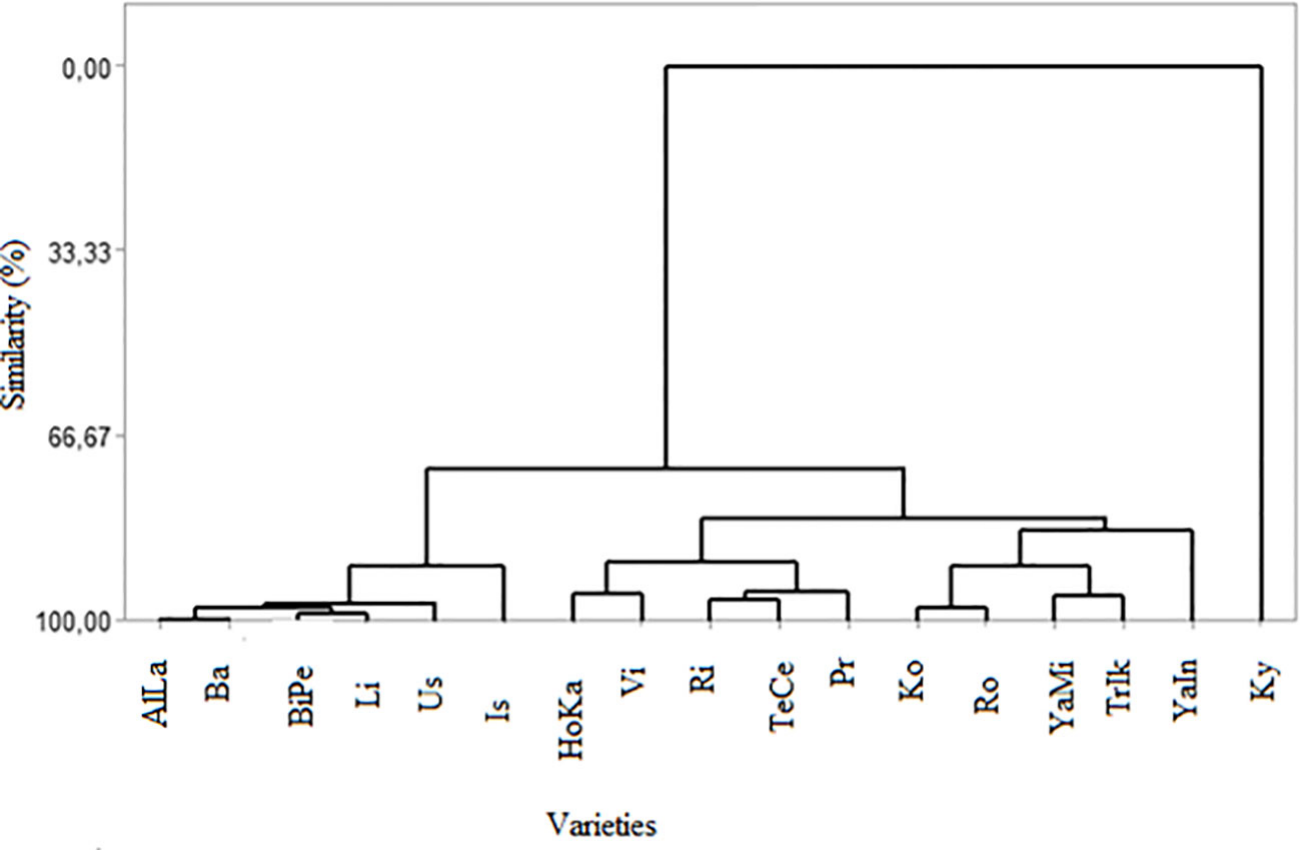
Dendrogram of the phenotypic similarities among the grape varieties in terms of organic acid compounds.

This study employed cluster analysis and multidimensional scaling to assess phenotypic similarities among the grape varieties based on their sugar and organic acid contents, as shown in **Figure 1** and **Table 3**. Statistical analysis indicated significant differences among grape varieties, either individually or in their interactions, across various parameters. The similarity dendrogram based on these contents revealed a high degree of similarity among 16 grape varieties, excluding AlLa and HoKa, with similarity levels ranging from 99.49% to 72.36%. The highest similarity was found between the AlLa and Ba varieties at 99.49%, followed by BiPe and Vi at 98.43%, AlLa and BiPe at 97.54%, and Ko and Ro at 97.3848%. The lowest similarity was observed among the AlLa, HoKa, and Ky varieties. In addition to the similarity analysis, cluster analysis was performed, resulting in three major groups of grape varieties. The first cluster included AlLa, Ba, BiPe, Li, Us, and Is. The second cluster comprised 10 varieties: Hoka, Vi, Ri, TeCe, Pr, Ko, Ro, YaMi, TrIk, and YaIn. Notably, the Ky variety formed a separate group distinct from the other two clusters.

**Table 3:** Phenotypic similarities (in percent) between varieties in terms of the organic acid compounds in the different grape varieties with cluster analysis and multidimensional scaling.

Step	No. of clusters	Similarity (%)	Joined cluster		New cluster	No. of varieties in cluster	Varieties
1	16	99.4991	1	2	1	2	1: AlLa
2	15	98.4371	3	8	3	2	2: Ba
3	14	97.5408	1	3	1	4	3: BiPe
4	13	97.3848	6	11	6	2	4: HoKa
5	12	96.7623	1	14	1	5	5:Is
6	11	96.0991	10	12	10	2	6: Ko
7	10	95.5140	9	13	9	2	7: Ky
8	9	95.1165	4	15	4	2	8: Li
9	8	94.6577	10	17	10	3	9: YaMi
10	7	90.0515	1	5	1	6	10: Ri
11	6	89.8368	6	9	6	4	11: Ro
12	5	89.1796	4	10	4	5	12: TeCe
13	4	83.6762	6	16	6	5	13: TrIk
14	3	81.3637	4	6	4	10	14: Us
15	2	72.3624	1	4	1	16	15: Vi
16	1	0.0000	1	7	1	17	16: YaIn
							17: Pr

## 4 Discussion

The diverse responses of the grape varieties cultivated under identical ecological conditions provided crucial insights into the interplay between genetic makeup and environmental factors in determining viticultural success. In our investigation of TSS content across varying maturation stages of the grape cultivars in Kalecik, we uncovered notable variability that points to a complex interplay of physiological, environmental, and genetic influences. TSS, an essential measure of grape ripeness and sugar content, varied significantly among the early-, mid-, and late-season varieties. This variability, potentially stemming from climatic and genetic factors, resonates with the insights provided by Jones (2005) and Bunting et al. (2021), who emphasized the impact of these factors on the quality characteristics of grapes. This study has promising implications for grape breeding programs. The observed TSS differences underscore the potential of selecting and breeding grape cultivars that are optimally adapted to specific microclimatic conditions. Understanding how environmental factors such as temperature and sunlight, as elucidated by Jones and Webb (2010), affect grape composition can guide breeders in choosing varieties that thrive under specific climatic conditions, thereby enhancing grape quality and yield. Genetic diversity, as highlighted by Mullins et al. (1992), plays a pivotal role in determining the sugar metabolism and ripening traits of a variety. Our findings revealed a broad spectrum of TSS values, suggesting a rich genetic tapestry within the Ankara-Kalecik grape varieties. This genetic variability offers a valuable resource for breeders aiming to enhance specific traits such as sugar accumulation rates (Varandas et al., 2004). For instance, the rapid sugar accumulation observed in the early-season varieties, such as YaMi, reaching up to 21.0°Brix, could be harnessed to develop new cultivars that achieve desired sugar levels more efficiently. Conversely, the lower TSS levels in varieties such as BiPe (14.1°Brix) indicate their potential for improving sugar accumulation processes through selective breeding. The significant differences (*p* ≤ 0.05) in the TSS contents across the two years further validated the reliability of these varietal characteristics and their suitability for targeted breeding strategies (**Table 1**). Palliotti et al. (2014) advocated vineyard management practices tailored to individual grape varieties. Our study extends this recommendation to grape breeding, suggesting the need for an integrated approach that considers physiological, environmental, and genetic aspects for the development of new grape varieties. Such an approach is crucial not only for optimizing grape quality but also for enhancing the overall efficiency and sustainability of grape production and winemaking. While our research provides foundational insights, further exploration is necessary to fully understand the causative factors behind TSS variability in grapes. Future studies should focus on detailed genetic analyses, alongside environmental assessments, to inform and refine breeding strategies. This comprehensive approach will be instrumental in advancing grape cultivation, meeting industrial demands, and responding to changing climatic conditions.

In our study, we found significant year-to-year variations in the °Brix values, underscoring the influence of annual environmental factors on grape quality. All varieties exceeded the 14°Brix threshold, indicating good sensory quality. This aligns with the findings of Bondada et al. (2017) and highlights the importance of harvest year in TSS content, echoing (Pavloušek and Kumšta, 2011). We also observed disparities in the total TA content across grape varieties and years. Varieties such as HoKa, TeCe, Ri, and Ky showed consistent TA values, with TeCe having the highest TA content. This variability is influenced by both the harvest year and grape variety, supporting the conclusions of Liu et al. (2006). The TSS/TA ratio, which is crucial for taste, varied significantly. In 2018, this ranged from 18.45 in TeCe to 38.65 in Vi and was from 17.36 in TrIk to 45.26 in Is in 2019. These variations are within the ideal range for grape juice, indicating that varieties such as Ba, Ko, YaMi, Ri, Vi, and Is have excellent taste profiles, as suggested by dos Santos Lima et al. (2014) and Xu et al. (2017). Regarding pH, a key determinant of quality, the highest value in 2018 was 3.6 (Us and YaIn) and the lowest was 3.0 (TeCe). In 2019, the highest value was found in Us, YaIn, and Ri (3.7), while the lowest was in TeCe and TrIk (3.4), in line with Zhang et al. (2021). These findings have significant implications for grape breeding and viticulture. Understanding the impact of environmental factors on key quality parameters, such as °Brix, TA, TSS/TA ratio, and pH, can guide the selection of grape varieties best suited to specific ecological conditions. This knowledge can be applied in breeding programs to develop varieties with desired taste profiles and quality characteristics tailored to varying environmental conditions and consumer preferences. On the other hand, the results of our study, focusing on the sugar content in grapes, particularly the fructose and glucose levels and their ratios, offer pivotal insights for grape breeding when viewed in light of existing scholarly research. The significant variation in the sugar content among the different grape varieties observed in our study corroborates the widely accepted notion that sugar accumulation in grapes is influenced by a complex interplay of genetic, environmental, and cultural factors (Liu et al., 2006). This is particularly evident in the varied glucose and fructose levels across varieties, emphasizing the role of genetic factors in grape sugar metabolism. Consistent with Liang et al. (2011), our findings confirm that glucose and fructose are the predominant sugars in ripe grapes, accounting for approximately 99% of the total sugars. This dominance is crucial for defining the taste and quality of grapes. The progression from glucose as the dominant sugar to a balance with fructose during ripening highlights the dynamic nature of sugar metabolism during grape development. Our results, showing significant differences in the total sugar content among varieties such as Ba, Ko, Us, and Li, align with Orak (2007) and underscore the genetic diversity in grape cultivars. This diversity offers the potential for selective breeding aimed at enhancing specific sugar profiles desirable in grapes. Furthermore, the correlation between grape maturity and sugar concentration has profound implications for grape quality, influencing phenolic compounds and the sugar-to-acid ratio, which are essential for the sensory profile of grapes and wine (Rusjan and Korosec-Koruza, 2007). Our findings are highly valuable for grape breeding. Understanding the genetic basis of sugar accumulation and its relationship with environmental factors will enable breeders to strategically select parent varieties for breeding programs. The objective of this study was to cultivate new grape varieties with optimal sugar content, thereby influencing their taste, quality, and consumer appeal. Such breeding efforts could lead to grapes being better adapted to specific environmental conditions and more aligned with market demands and consumer preferences. Thus, our study contributes significantly to the field of grape breeding, offering a direction for developing grape varieties with enhanced sugar profiles and overall quality.

In the context of grape breeding, the findings of our study on the organic acid concentrations in grape varieties, as presented in **Table 2**, offer intriguing prospects. We observed a substantial range in the total organic acid values, from 3.95 g/L in the Ro variety to 24.94 g/L in Ky (**Table 2**). This wide spectrum is in line with previous research on grape varieties and points to significant opportunities for grape breeders. The notable variation in the tartaric acid levels among the grape varieties, which was found to be statistically significant (*p* ≤ 0.05), is a key consideration for breeders. Keskin et al. (2021) observed that malic and tartaric acids, the major organic acids in grapes, show a broad range in concentration due to regional and varietal factors. This aligns with our findings, where the malic acid contents in 12 varieties were consistent with established values, whereas four cultivars, including Ky, had markedly higher levels. Such insights allow breeders to select parent plants for crossbreeding to achieve the desired balance of these acids, which is crucial for the taste and quality of the final grape product. The significant variability observed in the concentrations of citric and succinic acid among the grape varieties further emphasizes the potential for targeted breeding. The ranges of these acids in our study, which fall within the scope reported by Soyer et al. (2003) and Dutra et al. (2021), highlight the diverse acid profiles that can be obtained through careful selection and breeding. Our findings also draw attention to the unique case of oxalic acid, which was notably low in all varieties, except for Ky. This is particularly relevant given that other studies, such as that of Yinshan et al. (2017), have reported the absence of oxalic acid in many grape cultivars. Breeding strategies could potentially focus on this aspect to develop varieties with specific oxalic acid profiles. Moreover, the wide variation in the tartaric acid/malic acid (T/M) ratio across the grape varieties in our study, which aligns with the range found by Liu et al. (2006), opens up avenues for breeding grapes with specific acid ratios, catering to different wine styles or fresh consumption preferences. In addition, the substantial variations in the total organic acids that we observed underscore the potential for breeders to develop varieties with specific total acid content. This aspect is crucial as it impacts not only the sensory attributes of grapes but also their suitability for various end uses.

In the context of grape breeding, our use of cluster analysis and multidimensional scaling to evaluate phenotypic similarities among grape varieties based on their sugar and organic acid contents offers valuable insights for breeders. The analysis, as depicted in **Figure 1** and **Table 3**, showed a high degree of similarity among the 16 varieties, with notable exceptions of Al and HoKa. This high similarity, with levels ranging from 99.49% to 72.36%, suggests that these varieties share significant commonalities in their biochemical makeup, which could be leveraged in breeding programs. The highest similarity observed between AlLa and Ba (99.49%), followed by BiPe and Vi (98.43%) and AlLa and BiPe (97.54%), indicates the potential for the cross-breeding of these varieties to combine desirable traits. The distinct cluster of the Ky variety, separated from the other two main groups, highlights its unique genetic makeup, suggesting that it could be a valuable resource for introducing new traits into existing varieties. Cluster analysis, which categorized the 17 grape varieties into three distinct groups, can guide breeders in selecting varieties for specific breeding objectives. For example, the first cluster, comprising AlLa, Ba, BiPe, Li, Us, and Is, could be targeted for breeding programs aimed at certain wine styles or table grape characteristics, considering their similar sugar and organic acid profiles. These distinctions, likely influenced by regional conditions during the growing season, underscore the importance of environmental factors in grape development. As noted in previous studies, such as those by Liu et al. (2006), the sugar composition in grapes tends to be stable and less susceptible to climate-induced changes. However, other components, including organic acids, are significantly influenced by harvest conditions and climate, as well as by the genetic makeup of grape varieties (Yinshan et al., 2017). Therefore, understanding these variations and similarities among varieties is crucial for grape breeding. This allows breeders to make informed decisions about which varieties to cross for desired outcomes, considering both the genetic factors inherent in the varieties and the influence of environmental conditions on their development. This approach could lead to the creation of new grape varieties with optimized characteristics for specific purposes, whether for winemaking or fresh consumption, thus enhancing the diversity and quality of the grapes available to the industry.

## 5 Conclusions

The results indicate that the 17 table grape varieties cultivated in the Ankara-Kalecik province of Turkey exhibit significant concentrations of valuable organic acids and sugars with potential health benefits. This research underscores the substantial influence of grape varieties on the composition and content of sugars and organic acids within berries. Among the studied genotypes, Ba, HoKa, TeCe, YaMi, AlLa, Li, and Ri displayed higher levels of malic acid, whereas Ky exhibited superior performance in terms of all organic acid constituents. Notably, Ky stands out as a remarkable variety, characterized by having one of the highest total organic acid contents among the varieties and is particularly rich in tartaric, malic, citric, succinic, and oxalic acids. Furthermore, the findings revealed that the sugar concentrations in these 17 grape varieties exhibited greater stability than did the organic acids. It is important to note that the composition of sugar and organic acid compounds was significantly influenced by both genotype and harvest year. In summary, understanding the variations in sugar and organic acid accumulation among different grape varieties is of great significance for determining the overall flavor profile at the time of harvest.

## Data availability statement

The original contributions presented in the study are included in the article. Further inquiries can be directed to the corresponding authors.

## Author contributions

BK: Conceptualization, Data curation, Formal analysis, Investigation, Methodology, Project administration, Resources, Software, Supervision, Validation, Visualization, Writing – original draft, Writing – review & editing. OU: Conceptualization, Data curation, Formal analysis, Investigation, Software, Writing – review & editing. SK: Conceptualization, Data curation, Formal analysis, Investigation, Software, Resources, Validation, Visualization, Writing – review & editing. HH-V: Data curation, Formal analysis, Funding acquisition, Methodology, Resources, Visualization, Conceptualization, Investigation, Software, Writing – review & editing. OK: Conceptualization, Data curation, Formal analysis, Investigation, Software, Resources, Validation, Visualization, Writing – review & editing.
